# Experimental and Prediction Study of Displacement-Rate Effects on Flexural Behaviour in Nano and Micro TiO_2_ Particles-Epoxy Resin Composites

**DOI:** 10.3390/polym12010022

**Published:** 2019-12-20

**Authors:** George C. Papanicolaou, Aikaterini E. Manara, Lykourgos C. Kontaxis

**Affiliations:** The Composite Materials Group, Department of Mechanical Engineering & Aeronautics, University of Patras, Patras GR-26500, Greece; manarakaterina@gmail.com (A.E.M.); lykourgoskontaxis@gmail.com (L.C.K.)

**Keywords:** particulate, epoxy resin, nanocomposite, microcomposite, flexural behavior, displacement-rate, modelling, property prediction model (PPM), titanium dioxide, Titania

## Abstract

Epoxy resin composites with different weight fractions of TiO_2_ microparticles (1%, 5%, 10%, 15%, 20%) and of TiO_2_ nanoparticles (0.5%, 1%, 2%, 3%) were prepared. The particle size of the nanoparticles was averaged around 21 nm while the particle size of the micro TiO_2_ particles was averaged around 0.2 μm. The morphology of the manufactured particulate composites was studied by means of scanning electron microscopy (SEM). The mechanical properties of both nanocomposites (21 nm) and microcomposites (0.2 μm) were investigated and compared through flexural testing. Furthermore, the effect of displacement-rate on the viscoelastic behavior of composite materials was investigated. The flexural tests were carried out at different filler weight fractions and different displacement-rates (0.5, 5, 10, 50 mm/min). The influence of TiO_2_ micro- and nanoparticles on the mechanical response of the manufactured composites was studied. For micro TiO_2_ composites, a maximum increase in flexural modulus on the order of 23% was achieved, while, in the nanocomposites, plastification of the epoxy matrix due to the presence of TiO_2_ nanoparticles was observed. Both behaviors were predicted by the Property Prediction Model (PPM), and a fair agreement between experimental results and theoretical predictions was observed.

## 1. Introduction

In the past few decades, epoxy resin has been used in several applications, such as surface coatings, adhesives, painting materials as well as electronic devices. However, since epoxy resin is characterized by low mechanical properties in comparison to traditional structural materials, it is usually filled with several types of reinforcements. This modification affects amongst others, the deformation mechanism of the resulted composite. The main parameters affecting the mechanical properties of epoxy–matrix composites are the filler concentration, the adhesion of the filler to the epoxy matrix, the particles dispersion within the resin, as well as the shape and size of the reinforcing filler. In recent years, scientists attempted to use nanoparticles, as fillers, in an effort to increase the mechanical properties of the epoxy resin and not only. Nanoparticles, that are being used, are carbon nanotubes (CNTs), silica clay, SiO_2_, Al_2_O_3_, and TiO_2_ [1,2,3,4,5].

It has been observed that epoxy-nanocomposites mechanical properties are improved, as the filler–matrix adhesion is increased. In case of a strong interfacial bonding between polymer and particle, the nanocomposite mechanical properties strongly depend on the material density of the region surrounding the inclusion. On the other hand, the size of the particles plays an important role in improving the mechanical properties of the composite. The lower the filler diameter, the higher the filler surface/contact area is. Thus, in case of micron sized particles, the size of the low-density interfacial region of the pure polymer surrounding the inclusion is higher, so that the contribution of the filler is reduced, concluding that the mechanical properties of the composite will not be improved that much [6]. On the contrary, the nanoparticles can fill up the weak regions within the epoxy resin, and thus increasing the filler–matrix interaction force. The increase of the extent interfacial region can significantly improve the mechanical properties of the epoxy/nanocomposite. However, the nanoparticles have a difficulty to disperse into the matrix, so it is crucial to find the proper manufacturing technique to improve particle dispersion within the matrix material. A technique that is commonly applied is the ultrasonic homogenization; however, as every technique has its disadvantages. More precisely, this technique decreases the gelling time of the epoxy resin [7,8,9,10,11]. 

The mechanical response of particulate reinforced composites also depends on the rate of deformation. It was found that particulates increase the strain rate sensitivity concerning tensile modulus, but this sensitivity decreases when measuring yield strength [12]. The effect of strain rate on the flexural properties of some polymer matrix composites has been studied and, as it was found, the flexural modulus and flexural strength both increase linearly with the logarithm of the strain rate [13]. Furthermore, the strain-rate sensitivity seems to be slightly more pronounced in shear than in tension and compression modes [14]. However, the understanding of variation in deformation mechanism of particulate composites at different strain rates is still not investigated theoretically; therefore, there is no mathematical model for the prediction of material properties with variation in strain rate [15,16,17]. 

The TiO_2_ is extensively used in the industry as additives in plastics, agglomerates for thermal sprays, air/fuel ratio controller in automobile, attenuation of ultraviolet light, catalysts and catalyst supports, demilitarization of chemical and biological warfare agents, electrode materials in lithium batteries, energy converter in solar cells, gas sensors, inorganic membranes, photo catalytic degradation of bacteria and grime, photochemical degradation of toxic chemicals, piezoelectric capacitors, solid oxide fuel cell, UV protection, and waste water purification [18,19,20,21,22,23,24,25].

In the present study, epoxy resin reinforced with micro TiO_2_ and nano TiO_2_ were manufactured in order to investigate the effect of the size of the reinforcing particles on the mechanical and viscoelastic properties of the resulted composite materials. Applied titanium dioxide particles had two different diameters; namely: 200 nm for the microcomposites and 21 nm for the nanocomposites. A series of different displacement-rates (0.5, 5, 10, 50 mm/min) has been applied on composite specimens with different filler weight fractions subjected to three-point bending and subsequently experimental results were predicted by means of the Property Prediction Model (PPM) model.

As far as the authors are aware, although several works have been already published concerning TiO_2_/epoxy composites, the proposed study offers a comparison between TiO_2_ particles-epoxy resin micro- and nanocomposites, taking into consideration the particle–matrix interfacial phenomena. In addition, the application of the recently improved Property Prediction Model (PPM) to predict the composite behavior by using only two experimental data points constitutes unique and novel aspects that are of importance for understanding the difference between micro- and nanocomposites mechanical and viscoelastic behavior. Furthermore, the presented modelling gives a detailed description and a physical meaning concerning the interrelation existing between structure, filler dimension, extent of filler loading, and macroscopic behavior.

## 2. Materials and Methods 

### 2.1. Materials

Epoxy resin RenLam CY219 (Bisphenol A) combined with a curing agent HY 5161 (Diamine) at a ratio 2:1 by weight was used as matrix material. Gelling time was 24 h at 50 °C, and mass density of cured polymer 1.1 g·cm^−3^. Viscosity of the system CY219 and HY 5161 was 1–1.2 Pas at 25 °C. Titanium(IV) oxide nano-powder supplied by Sigma-Aldrich (MO, USA) was used, with an average particle size of 21 nm, specific surface area 35–65 m^2^·g ^−1^, mass density 4.26 g·cm^−3^, and purity of TiO_2_ nanoparticles ≥99.5%, exposed for thermal treatment at 50 °C for 24 h to ensure discard of H_2_O molecules absorbed by TiO_2_ nanoparticles. Titanium dioxide Rutile 2902 microparticles supplied by Vellis Chemicals (Thessaloniki, Greece) were used, with an average particle size of 0.2 μm and mass density 4.1 g·cm^−3^, also exposed for thermal treatment at 50 °C for 24 h. The physical properties of TiO_2_ micro- and nanoparticles are presented in Table 1 as given both by the manufacturers’ datasheets and literature [26].

### 2.2. Micro-Particulate Specimens Manufacturing

Titanium dioxide was placed in an oven at 50 °C for 24 h in order to dry, as mentioned above. Next, the resin was placed in an oven for 10 min at 40 °C in order to decrease its viscosity. Polymer resin and TiO_2_ microparticles 0.2 μm in size (1, 5, 10, 15 wt.%) were carefully mixed by means of an electrical stirrer, in proper quantities, in order to achieve uniform distribution of the fillers into the matrix. Then, the mixture was placed in a vacuum chamber for 5–6 min to reduce the amount of entrapped air. The mixture was then poured in a proper metallic mould and subsequently cured in an oven at 50 °C for 24 h. 

### 2.3. Nano-Particulate Specimens Manufacturing

The resin was placed in an oven for 10 min at 40 °C in order to decrease its viscosity. TiO_2_ nanoparticles were added to the hardener in a plastic beaker and mixed by means of an electrical stirrer for preliminary dispersion. The mixture was placed in the sonicator for 5 min at 10 kHz. In order to avoid a temperature increase during sonication, external cooling was employed by submerging the mixing beaker into a mixture of ice and saltwater. The beaker with the resin was also submerged into a mixture of ice and saltwater and the TiO_2_-hardener system was added in the resin and mixed via an electrical stirrer (JJ-1, Jiangsu, China) until homogeneity was achieved. The final mixture was placed in a vacuum chamber for 5–6 min to reduce the amount of entrapped air. Finally, the mixture was poured into a proper metallic mould and cured in an oven at 50 °C for 24 h.

Bandelin Sonopulse HD 2200 sonicator homogenizer (Bandelin, Berlin, Germany) was used for dispersion of the TiO_2_ nanoparticles. The maximum power output was 200 W applied at a maximum frequency of 20 kHz using a 13 mm diameter titanium flat tip.

### 2.4. Experimental Characterization

The degree of dispersion of TiO_2_ particles into the epoxy matrix was checked by means of a Scanning Electron Microscopy (SEM device, Model SUPRA 35VP, Zeiss, Jena, Germany) in the absence of any conductive sputtering.

Preliminary flexural properties of the materials manufactured were determined in accordance to ASTM D 790-03 with a displacement-rate of 1 mm/min (Instron 4301). 

Next, flexural displacement-rate tests conducted by applying four different displacement-rates (0.5, 5, 10 and 50 mm/min) (Instron 4301, High Wycombe, UK). 

All specimens used for the flexural tests had dimensions 100 × 12.8 × 2.5 mm and a span length of 63 mm. The specimens were manufactured in accordance to ASTM D 790-03. Five or more specimens per each filler weight fraction were tested to insure the repeatability of results.

### 2.5. Theoretical Backgraound

#### Property Prediction Model (PPM)

The Property Prediction Model (PPM) has been developed, by the first author, aiming to predict the composite property-value variation as a function of the filler concentration (*C_f_*) in particulate composites. The present model is the improved version of the Modulus Prediction Model (MPM) already presented in [27,28] after it was found that it can predict a series of different material properties in addition to the elastic modulus [29]. For the model application, only two experimental points are needed. The first experimental point (*C*_1_, *P*_1_) should reflect the composite behavior at very low filler concentration, while the second one, (*C*_2_, *P*_2_) should represent composite behavior at a high filler concentration. This is because, at very low *C_f_*, composite behavior is mainly dominated by the filler–matrix adhesion, while, at high *C_f_*, composite behavior is mainly affected by the filler dispersion within the matrix material. 

The main assumption of the model is that the property variation of the composite with filler-concentration can be described by a second order polynomial function, as:

(1) $$P_{c}=AC_{f}^{2}+BC_{f}+P_{m},$$

where *P_c_* and *P_m_* represent the composite and matrix property value, respectively. Given two experimental points (*C*_1_, *P*_1_) and (*C*_2_, *P*_2_), we get:

(2) $$P_{1}=AC_{1}^{2}+BC_{1}+P_{m},$$

(3) $$P_{2}=AC_{2}^{2}+BC_{2}+P_{m}.$$

Solving these equations for *A* and *B* results in:

(4) $$A=\frac{P_{2}-P_{m}}{C_{2}\left(C_{2}-C_{1}\right)}-\frac{P_{1}-P_{m}}{C_{1}\left(C_{2}-C_{1}\right)},$$

(5) $$B=\frac{\left(P_{1}-P_{m}\right)C_{2}}{C_{1}\left(C_{2}-C_{1}\right)}-\frac{\left(P_{2}-P_{m}\right)C_{1}}{C_{2}\left(C_{2}-C_{1}\right)}.$$

Setting now,

(6) $$\lambda =\frac{P_{2}-P_{m}}{P_{f}C_{2}\left(C_{2}-C_{1}\right)},$$

(7) $$\kappa =\frac{P_{1}-P_{m}}{P_{f}C_{1}\left(C_{2}-C_{1}\right)}.$$

*A* and *B* can be written as:

(8) $$A=\left(\lambda -\kappa \right)P_{f},$$

(9) $$B=\left(\kappa C_{2}-\lambda C_{1}\right)P_{f}.$$

Since *κ* depends on the low filler concentration (*C*_1_) property value (*P*_1_), i.e., point (*C*_1_, *P*_1_), where composite behavior is mainly dependent upon the filler–matrix adhesion, the κ-parameter is named the adhesion coefficient. Similarly, since *λ* depends on the high-filler concentration (*C*_2_), property value (*P*_2_), i.e., point (*C*_2_, *P*_2_), where composite behavior is mainly dependent upon the filler dispersion, the *λ* parameter is called the dispersion coefficient.

We are now introducing the reduced parameters, *L* and *K*, as follows:

(10) $$L=\frac{\lambda }{\lambda +\kappa },$$

(11) $$K=\frac{\kappa }{\lambda +\kappa }.$$

Since *L* is proportional to *λ,* which is the dispersion coefficient, it represents the percentage contribution of the filler dispersion within the matrix material on the overall composite behavior, and it is called degree of dispersion. Similarly, since *K* is proportional to *κ*, which is the filler–matrix adhesion coefficient, it represents the percentage contribution of the filler–matrix adhesion on the overall composite behavior, and it is named degree of adhesion.

It is clear that

(12) L+K=1.

In addition, if

(13) $$\delta_{1}=P_{1}-P_{m},$$

(14) $$\delta_{2}=P_{2}-P_{m},$$

then

(15) $$L=\frac{\delta_{2}}{\delta_{2}+\delta_{1}\left(C_{2}/C_{1}\right)},$$

(16) $$K=\frac{\delta_{1}}{\delta_{1}+\delta_{2}\left(C_{1}/C_{2}\right)},$$

and

(17) $$\frac{K}{L}=\frac{\left(\delta_{1}/\delta_{2}\right)}{\left(C_{1}/C_{2}\right)}=\frac{\lambda }{\kappa }.$$

## 3. Results and Discussion

### 3.1. Scanning Electron Microscopy (SEM)

**Figure 1:**
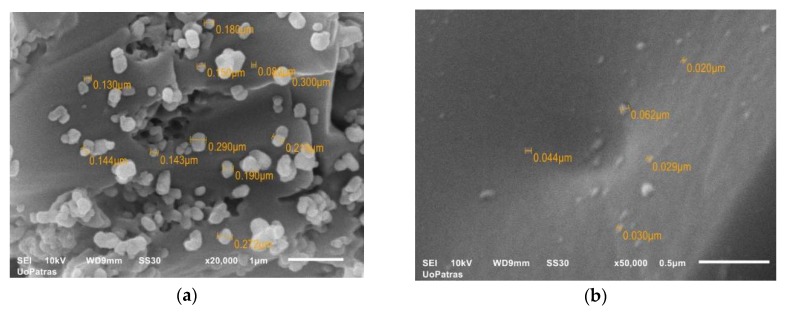
SEM photo- micrographs of TiO_2_/epoxy particulate composites at: (**a**) 20 wt.% microparticles; (**b**) 3 wt.% nanoparticles of TiO_2_.

The surface topography of micro- and nano-epoxy/TiO_2_ composites can be observed through scanning electron microscopy, in Figure 1a,b. The SEM image presented in Figure 1a corresponds to the highest TiO_2_ microparticles weight fraction (20 wt.%). As far as microparticles are concerned, the formation of aggregates can be detected, since this is the maximum weight fraction, above which TiO_2_ microparticle aggregation is inevitable. As the markers on the image indicate, the size of the particles varies from 0.13 μm to 0.3 μm.

Figure 1b shows the 3 wt.% TiO_2_ nanocomposite. It is difficult to clearly observe the nanoparticles because of their extremely small size (21 nm). Furthermore, there were not any large visible agglomerations, concluding that the particle distribution was a satisfactory one. As the markers on the image indicate, the size of the particles varies from 0.02 μm to 0.062 μm.

### 3.2. Flexural Characterization

**Figure 2:**
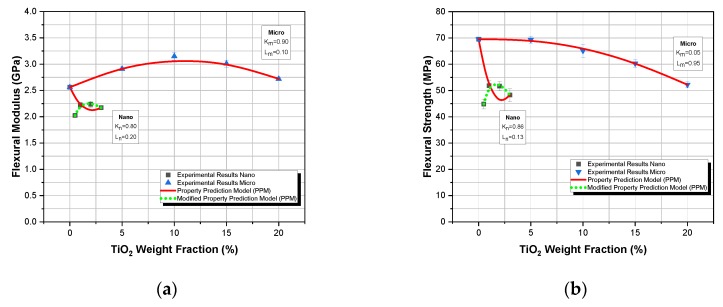
Comparison between experimental values and theoretical predictions as derived from the property prediction model (PPM) for the flexural properties (**a**) flexural modulus and (**b**) flexural strength versus TiO_2_ weight fraction of the TiO_2_ micro and nanoparticle-epoxy matrix composites investigated.

The materials manufactured were then initially characterized in three-point bending tests in an Instron 4301 universal testing machine. The variation of the flexural modulus and the flexural strength of the composites investigated with the filler–weight fraction for both types of composites are shown in Figure 2. In addition, the Property Prediction Model (PPM) modulus predictions were plotted together with the respective experimental data for comparison. 

As shown in Figure 2a, the model predicts extremely well the variation of the flexural modulus as a function of the filler–weight fraction, for TiO_2_ microcomposites and sufficiently good for TiO_2_ nanocomposites. In the case of microcomposites, as the filler–weight fraction increases, an initial increase in flexural modulus is observed, and this is attributed to the matrix reinforcement provided by the TiO_2_ microparticles. However, with further increase in filler–weight fraction, and for weight fractions higher than 10%, a decrease in flexural modulus is detected. The observed decrease in flexural modulus is attributed to the formation of agglomerations that are visible in Figure 1a.

On the contrary, nanoparticle TiO_2_ composites do not exhibit a similar behavior to their micro-particulate counterparts. Initially, a decrease is observed in flexural modulus, which results in modulus values lower than this of the pure resin—a behavior previously encountered [30]. As the size of nanoparticles approaches, the molecular size of polymeric chains, the nanoparticles interfere with polymer macromolecules, delaying or even prohibiting the crosslinking mechanism between polymer chains to take place. Therefore, instead of reinforcing the matrix, a plastification effect is taking place leading to a reduction in flexural modulus [31,32,33]. However, after the initial drop of the modulus, as the TiO_2_ nanoparticles weight fraction increases, a similar behavior to that of the micro-particle composites can be observed; i.e., an initial increase which appears to “reinforce” the now plasticised matrix, and a small decrease attributed to the initiation of agglomerations formation. This behavior of the nano-particulate composites is observed for all the different displacement-rates applied. Thus, due to the matrix plastification effect observed, the 0.5% filler weight fraction could be considered as the initial point for the thus plasticized matrix. Therefore, if one considers the plastification effect of the matrix due to the nano-filler addition, then the Property Prediction Model (PPM) perfectly predicts all the values. 

For the TiO_2_ microcomposites, the deviation between experimental and theoretical values never exceeded 3.1%. A 0.80 degree of adhesion, *K*, was found for the nanocomposites while a 0.90 degree of adhesion for the TiO_2_/epoxy microcomposites was calculated. At this point, the distinction between wetting and adhesion must be pointed out. More precisely, in the present case, although, as it is well known, by decreasing particle diameter, the contact surface area increases, and it can be observed that the degree of adhesion in nanocomposites is lower to that in microcomposites. Taking into account that the degree of adhesion *K* reflects the adhesion contribution to the overall composite behavior, the above calculated values seem to be quite high for both types of composites. On the contrary, the degree of dispersion, *L*, reflects the dispersion contribution to the overall composite behavior, the model predicts a low value of 0.10 for the degree of dispersion of microcomposites, and a median degree of dispersion value equal to 0.20 for the nanocomposites. It must be stressed that *K* and *L* values are both property dependent and the above-mentioned values refer to the flexural modulus of the composites considered.

Next, concerning the strength variation shown in Figure 2b, a continuous decrease in strength with a filler–weight fraction is observed in the case of microcomposites while a perfect prediction was achieved. On the contrary, in the case of nanocomposites, an initial decrease of the strength is observed followed by a subsequent increase. However, if one considers the plastification effect of the matrix due to the nano-filler addition, then the model yet again perfectly predicts all the values. In the same figure, corresponding *K* and *L* values are shown for both nano and microcomposites having the same physical meaning already mentioned above.

**Figure 3:**
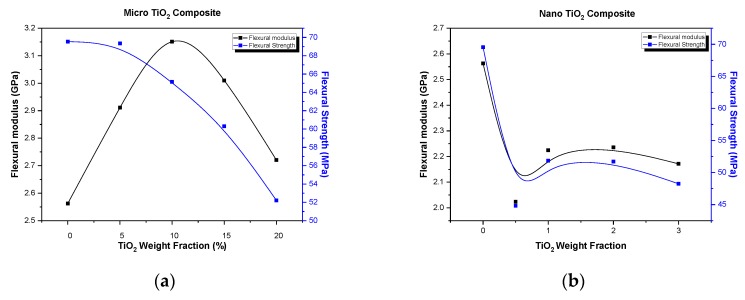
Comparison between the flexural modulus and the flexural strength variation with filler concentration: (**a**) micro-TiO_2_ particle-epoxy matrix composites; (**b**) nano-TiO_2_ particle-epoxy matrix composites.

In Figure 3, a comparison of the between flexural modulus and flexural strength variation vs. filler–weight fraction for micro- and nanocomposites is presented. As shown in Figure 3a, a continuous decrease in flexural strength of the composite is observed, as the micro-filler weight fraction is increased. This is a commonly observed behavior attributed to the voids, impurities, and particle aggregates developed due to the addition of particles. All the above-mentioned initiate micro-cracks and promote initiation of crack propagation, which inevitably reduces the flexural strength of the TiO_2_ microcomposites manufactured. From the same figure, we can observe a totally different way of variation between the modulus and the strength with filler concentration.

On the contrary, as shown in Figure 3b, nanocomposites exhibit the same trend of variation for the strength and the modulus. This type of behavior can be explained by the fact that, in the case of nanocomposites, as already mentioned above, filler addition to the matrix plasticizes the matrix leading to a decrease in modulus, while, at the same time, due to their bad dispersion into the matrix, introduce the creation of voids and aggregates leading to a decrease in strength. 

### 3.3. Displacement-Rate Results

The effect of displacement-rate on the viscoelastic behavior of composite materials was also investigated. The flexural tests were carried out at different filler weight fractions and different displacement-rates (0.5, 5, 10, 50 mm/min). In this investigation, the aim was to evaluate the effect of different particle size and different displacement-rates on the viscoelastic behavior of the particulate composites that were prepared. 

**Figure 4:**
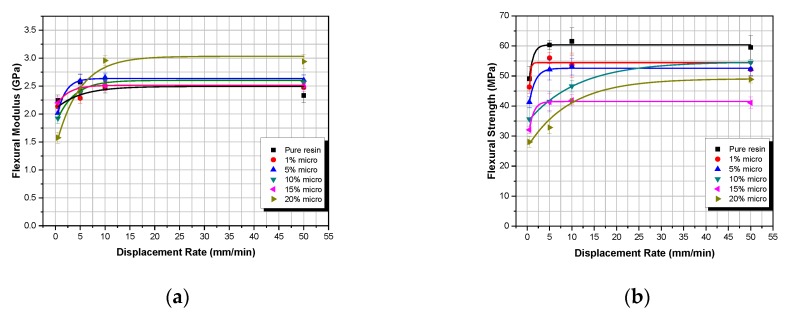
Microcomposites flexural properties versus displacement-rate for different filler–weight fractions: (**a**) flexural modulus; (**b**) flexural strength.

**Figure 5:**
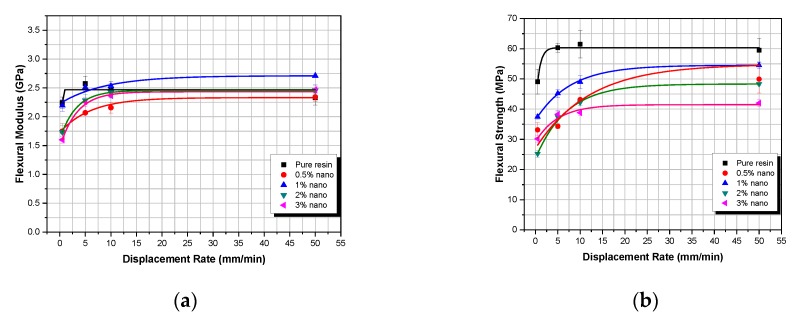
Nanocomposites flexural properties versus displacement-rate for different filler–weight fractions: (**a**) flexural modulus; (**b**) flexural strength.

In Figure 4 and Figure 5, the flexural properties versus displacement-rate with respect to weight fraction are given for both micro- and nanocomposites, respectively. 

As shown in Figure 4a,b, both flexural modulus and flexural strength in micro-particulate TiO_2_ composites show an initial increase with displacement-rate, until they both reach a plateau at the threshold displacement-rate of 5 mm/min. This can be attributed to inertia phenomena [34]. More precisely, as the displacement-rate increases, the TiO_2_ composite materials manufactured are unable to absorb the whole energy provided to them, so that both the flexural modulus and flexural strength reach their respective plateau (maximum values).

In Figure 5, the same behavior is observed as in Figure 4 for the TiO_2_ nano-particulate composites. As the displacement-rate is increasing, the flexural modulus and the flexural strength are also increasing until they reach a certain plateau. However, for the nanocomposites, the displacement-rate threshold is now located at 10 mm/min.

**Figure 6:**
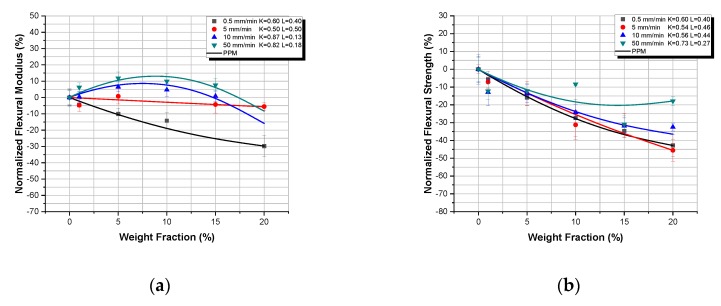
TiO_2_-Epoxy microcomposites: Normalized flexural properties versus TiO_2_ weight fraction and respective property prediction model (PPM) predictions, for different displacement-rates: (**a**) normalized flexural modulus; (**b**) normalized flexural strength.

**Figure 7:**
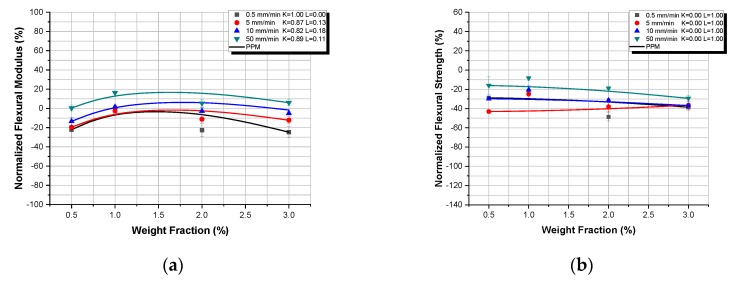
TiO_2_-Epoxy nanocomposites: Normalized flexural properties versus TiO_2_ weight fraction and respective property prediction model (PPM) predictions, for different displacement-rates: (**a**) normalized flexural modulus; (**b**) normalized flexural strength.

In Figure 6 and Figure 7, the flexural properties, normalized with respect to the respective matrix properties, for the micro- and nanocomposites respectively, versus TiO_2_ weight fraction, at different displacement-rates are presented. In addition, in the same figures, the Property Prediction Model (PPM) predictions are shown as continuous lines. 

In Figure 6a, at the lowest displacement-rate, the flexural modulus of TiO_2_ microcomposites continuously decreases, while it gradually increases as the displacement-rate increases. For the displacement rates of 0.5 and 5 mm/min, an almost linear behavior can be observed; for the 10 and 50 mm/min displacement-rates, by increasing the filler–weight fraction, an initial increase in flexural modulus is observed, followed by a subsequent decrease of the flexural modulus for filler–weight fraction higher than 10%. This is a recurring behavior that is also observed and explained in Section 3.2 as well as in literature [30]. As already mentioned, the initial increase in flexural modulus is attributed to the matrix reinforcement provided by the TiO_2_ microparticles. However, with further increase in filler–weight fraction, and after the same weight fraction threshold of 10%, as seen above in Section 3.2, a decrease in flexural modulus is detected, which is connected to the formation of agglomerations. 

The flexural strength, however, is decreasing with the increase of filler–weight fraction for all displacement-rates, a behavior observed and explained already in Section 3.2. Voids, impurities, and particle aggregates introduced along with the TiO_2_ particles initiate micro-cracks and promote crack propagation, which inevitably reduce the flexural strength of the TiO_2_ microcomposites manufactured. 

Finally, it is evident from both Figure 6a,b that the Property Prediction Model (PPM) predicts extremely well the variation of the flexural modulus as well as of flexural strength as a function of the filler–weight fraction, for TiO_2_ microcomposites, with minimal deviations.

Figure 7 shows the flexural modulus and the flexural strength variation with filler–weight fraction of TiO_2_-Epoxy nanocomposites for different displacement-rates. From these figures, it becomes clear that, due to the initial reduction observed in both properties upon adding even a small amount of nano-filler, the reduced properties (*P_c_* − *P_m_*)/*P_m_* attain negative values. In addition, with the exception of the 50 mm/min displacement-rate, in all the rest displacement rates, the flexural properties are lower than those of the pure resin. In that unique case, flexural moduli for different weight fraction are higher than the pure resin’s modulus, which is attributed to the high displacement-rate and the inertia phenomena following this and already discussed above. As explained, in Section 3.2, the small size of the nanoparticles interferes with polymer macromolecules, delaying or even prohibiting the crosslinking mechanism between polymer chains to take place. Therefore, nanoparticles instead of reinforcing the matrix plasticize the matrix leading to the reduction of the flexural properties (modulus and strength). However, after the initial drop of modulus, as the weight fraction of TiO_2_ nanoparticles is increased, a similar behavior to that of the micro particle composites can be observed; i.e., an initial increase which appears to “reinforce” the now plasticized matrix, followed by a subsequent decrease attributed to agglomerations.

**Figure 8:**
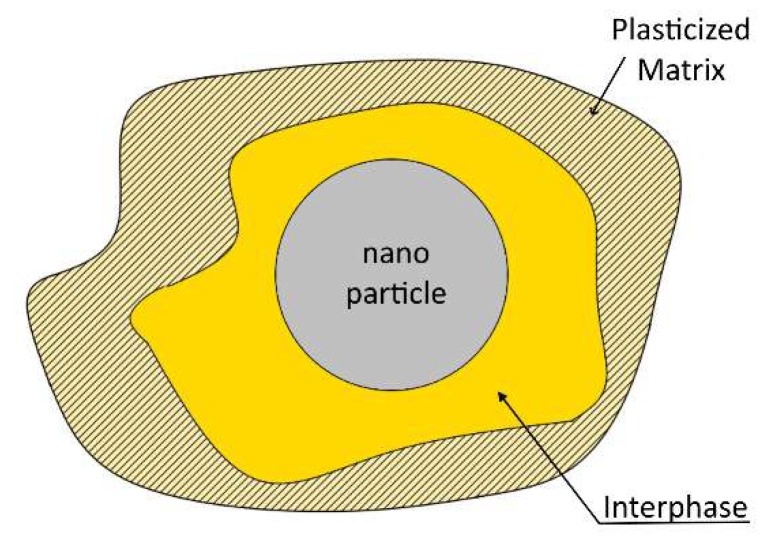
Graphical representation of the interphase region and the plasticized matrix.

Now, concerning respective Property Prediction Model (PPM) predictions, it is obvious from both figures that a fair agreement between experimental values and respective predictions was achieved. However, it must be pointed out that, for the above prediction, the matrix plastification effect has been always considered, according to which even with the addition of a very small amount of nano-fillers, matrix mechanical properties are abruptly reduced rendering the matrix material to behave differently when compared to the virgin one. Thus, according to these findings, one should take into account this phenomenon when studying epoxy–matrix nanocomposites with particle diameters smaller than 20 nm, taking as matrix properties those corresponding to the nanocomposites reinforced by an amount of 0.5 wt.% of filler and not those corresponding to the virgin material. These observations confirm the fact that resins behave differently as composite matrices and as a virgin material. The main reason for such a behavior is the existence of an interphase material created in the region at the close vicinity of each particle, with intermediate properties between those of the matrix material and the reinforcement. These properties are gradually varied from those of the inclusion to those of the matrix. The extent of such an interphase, depends not only on the nature of the constituent materials, but also on the filler fraction and the property studied at the time (Figure 8). Especially in the case of nanocomposites, due to the high filler–matrix contact area, almost all of the matrix material is transformed into “modified matrix”; i.e., interphasial material [35,36,37,38,39].

## 4. Conclusions

In the present study, the effect of the size of TiO_2_ micro- and nanoparticles, filler–weight fraction, and flexural displacement rate on the quasi-static mechanical properties of TiO_2_-Epoxy composites were investigated. Experimental results were also predicted through the application of the Property Prediction Model (PPM). The mechanical properties of both nano- and microcomposites were investigated and compared through flexural testing. The flexural tests were carried out at different filler weight fractions and different displacement-rates (0.5, 5, 10, 50 mm/min). Thus, the main conclusions derived in the present study are:

- Adding micro TiO_2_ in the epoxy matrix led to a decrease in flexural modulus at the low displacement-rate. As the displacement-rate increases, the flexural modulus increases with the increase of filler weight fraction [40,41,42].
- For the micro TiO_2_ composites, the flexural strength decreases with the increase of filler weight fraction at all strain rates applied, this behavior being in accordance with similar data found in literature. The decrease in flexural strength can be attributed to the imperfections and the voids introduced into the matrix with the addition of micro-particles.
- Adding nano TiO_2_ in the epoxy matrix led to a decrease in the flexural modulus of all nanocomposites tested under all strain rates applied, except of the highest strain rate. Such a behavior can be attributed to the fact that as the size of nanoparticles approaches the molecular size of polymeric chains, instead of reinforcing the matrix they interfere with polymer macromolecules and thus delaying the crosslinking mechanism between polymer chains to take place. In addition, the creation of a filler–matrix interphase with intermediate properties affects the matrix as well as the overall nanocomposite properties.
- The flexural strength decreases with the increase of filler weight fraction at all strain rates, for the TiO_2_ nanocomposites. The decrease in flexural strength can be attributed to the imperfections and the voids introduced when the nanoparticles are added.
- The nano TiO_2_ seems to deteriorate the flexural properties of the epoxy resin. The material seems to have a rubber-like behavior, which means that it tends to develop larger deformations at lower stresses.
- The Property Prediction Model predicted extremely well the mechanical response of both micro- and nanocomposites for all values of TiO_2_ weight fractions and all displacement rates applied with a minimum deviation from experimental findings.
- Finally, through the application of the Property Prediction Model (PPM) and for any mechanical property, it is possible to accurately calculate the percentage contribution of the filler–matrix adhesion (degree of adhesion *K*) as well as of the filler dispersion into the matrix material (degree of dispersion, *L*). *K* and *L* values along with the interphasial considerations can give a better insight into understanding the overall mechanical and viscoelastic (displacement rate effects) of both micro- and nanocomposites.

## Figures and Tables

**Table 1:** Physical properties of TiO_2_ micro- and nanoparticles.

Property	TiO_2_ Nanoparticles	TiO_2_ Microparticles
Particle size	21 nm	0.2 μm
Molar Mass	79.86 g/mol	79.86 g/mol
Specific Surface Area	35–65 m^2^/g (BET)	12 m^2^/g (BET)
Density	4.26 g/cm^3^	4.1 g/cm^3^
Melting point	1843 °C	1843 °C

## References

[1] Al-Ajaj I.A., Abd M.M., Jaffer H.I. (2013). Mechanical Properties of Micro and Nano TiO2/Epoxy Composites. Int. J. Min. Met. Mech. Eng..

[2] Al-Turaif H.A. (2010). Effect of nano TiO2 particle size on mechanical properties of cured epoxy resin. Prog. Org. Coat..

[3] Chatterjee A., Islam M.S. (2008). Fabrication and characterization of TiO2–epoxy nanocomposite. Mater. Sci. Eng. A.

[4] Ng C.B., Schadler L.S., Siegel R.W. (1999). Synthesis and mechanical properties of TiO2-epoxy nanocomposites. Nanostruct. Mater..

[5] Wetzel B., Rosso P., Haupert F., Friedrich K. (2006). Epoxy Nanocomposites—Fracture and Toughening Mechanisms, Eng. Fract. Mech..

[6] Ahmad F.N., Jaafar M., Palaniandy S., Azizli K.A.M. (2008). Effect of particle shape of silica mineral on the properties of epoxy composites. Compos. Sci. Technol..

[7] Kim B.C., Park S.W., Lee D.G. (2008). Fracture toughness of the nano-particle reinforced epoxy composite. Compos. Struct..

[8] Ho M.W., Lam C.K., Lau K.T., Ng D.H., Hui D. (2006). Mechanical properties of epoxy-based composites using nanoclays. Compos. Struct..

[9] Zhao R., Luo W. (2008). Fracture surface analysis on nano-SiO2/epoxy composite. Mater. Sci. Eng. A.

[10] Kurdi A., Wang H., Chang L. (2017). Effect of nano-sized TiO2 addition on tribological behaviour of poly ether ether ketone composite. Tribol. Int..

[11] Guo X., Guo Q., Nie J., Liu Z., Li Z., Fan G., Xiong D., Su Y., Fan J., Zhang D. (2018). Particle size effect on the interfacial properties of SiC particle-reinforced Al-Cu-Mg composites. Mater. Sci. Eng. A.

[12] Zhou Y., Mallick P.K. (2002). Effects of temperature and strain rate on the tensile behavior of unfilled and talc-filled polypropylene. Part I: Experiments. Polym. Eng. Sci..

[13] Zabihzadeh S.M. (2010). Flexural properties and orthotropic swelling behaviour of bagasse/thermoplastic composites. Bioresources.

[14] Delhaye V., Clausen A.H., Moussy F., Othman R., Hopperstad O.S. (2011). Influence of stress state and strain rate on the behaviour of a rubber-particle reinforced polypropylene. Int. J. Impact. Eng..

[15] Reis P.N.B., Gorbatikh L., Ivens J., Lomov S.V. (2019). Strain-rate sensitivity and stress relaxation of hybrid self-reinforced polypropylene composites under bending loads. Compos. Struct..

[16] Zaiemyekeh Z., Liaghat G.H., Ahmadi H., Khan M.K., Razmkhah O. (2019). Effect of strain rate on deformation behavior of aluminum matrix composites with Al2O3 nanoparticles. Mater. Sci. Eng. A.

[17] Prakash C., Emre Gunduz I., Oskay C., Tomar V. (2018). Effect of interface chemistry and strain rate on particle-matrix delamination in An Energetic Material. Eng. Fract. Mech..

[18] Cano L., Di Mauro A.E., Striccoli M., Curri M.L., Tercjak A. (2014). Optical and Conductive Properties of As-Synthesized Organic-Capped TiO2 Nanorods Highly Dispersible in Polystyrene-block-poly (methyl methacrylate) Diblock Copolymer. ACS Appl. Mater. Interfaces.

[19] Li Y., Luo J., Hu X., Wang X., Liang J., Yu K. (2015). Fabrication of TiO2 hollow nanostructures and their application in Lithium ion batteries. J. Alloy. Compd..

[20] Kociubczyk A.I., Vera M.L., Schvezov C.E., Heredia E., Ares A.E. (2015). TiO2 Coatings in Alkaline Electrolytes Using Anodic Oxidation Technique. Procedia Mater. Sci..

[21] Madhusoodana C.D., Manjunath S.P., Das R.N. (2012). Preparation of TiO2 membranes on silicon carbide supports for water filtration applications. Procedia Eng..

[22] Plecenik T., Moško M., Haidry A.A., Ďurina P., Truchlý M., Grančič B., Gregor M., Roch T., Satrapinskyy L., Moskova A. (2015). Fast highly-sensitive roomtemperature semiconductor gas sensor based on the nanoscale Pt–TiO2–Pt sandwich. Sens. Actuators B Chem..

[23] Choi K.I., Lee W., Lee S.H., Lim C. (2015). Synthesis of hierarchical hollow electrospun TiO2 nanofibers. Mater. Lett.

[24] Binu K.G., Shenoy B.S., Rao D.S., Pai R. (2014). A variable viscosity approach for the evaluation of load carrying capacity of oil lubricated journal bearing with TiO2 nanoparticles as lubricant additives. Procedia Mater. Sci..

[25] Purniawan A., French P.J., Pandraud G., Sarro P.M. (2010). TiO2 Ald Nanolayer as Evanescent Waveguide for Biomedical Sensor Applications. Procedia Eng..

[26] Kosmulski M. (2009). Surface Charging and Points of Zero Charge.

[27] Papanicolaou G.C., Koutsomitopoulou A.F., Sfakianakis A. (2011). Effect of thermal fatigue on the mechanical properties of epoxy matrix composites reinforced with olive pits powder. J. Appl. Polym. Sci..

[28] Papanicolaou G.C., Kontaxis L.C., Koutsomitopoulou A.F., Zaoutsos S.P. (2015). Stress relaxation behavior of starch powder-epoxy resin composites. J. Appl. Polym. Sci..

[29] Rabbi F. Ph.D. Thesis. http://scholarcommons.sc.edu/etd/2868.

[30] Papanicolaou G.C., Kostopoulos V., Kontaxis L.C., Kollia E., Kotrotsos A. (2018). A comparative study between epoxy/Titania micro-and nanoparticulate composites thermal and mechanical behavior by means of particle–matrix interphase considerations. Polym. Eng. Sci..

[31] Carponcin D., Dantras E., Dandurand J., Aridon G., Levallois F., Cadiergues L., Lacabanne C. (2014). Discontinuity of physical properties of carbon nanotube/polymer composites at the percolation threshold. J. Non-Cryst. Solids.

[32] Fu Y.F., Li J., Zhang F.Q., Xu K. (2016). The preparation and the friction and wear behaviours of TiO2/CNT/PI composite film. J. Exp. Nanosci..

[33] Abbasi H., Antunes M., Velasco J. (2018). Effects of Carbon Nanotubes/Graphene Nanoplatelets Hybrid Systems on the Structure and Properties of Polyetherimide-Based Foams. Polymers.

[34] Jacob G.C., Starbuck J.M., Fellers J.F., Simunovic S., Boeman R.G. (2004). Strain rate effects on the mechanical properties of polymer composite materials. J. Appl. Polym. Sci..

[35] Papanicolaou G.C., Michalopoulou M.V., Anifantis N.K. (2002). Thermal stresses in fibrous composites incorporating hybrid interphase regions. Compos. Sci. Technol..

[36] Papanicolaou G.C., Anifantis N.K., Keppas L.K., Kosmidou T.V. (2007). Stress analysis of short fiber-reinforced polymers incorporating a hybrid interphase region. Compos. Interfaces.

[37] Papanicolaou G.C., Keppas L.K., Anifantis N.K. (2008). Finite element prediction of debonding onset in short fiber reinforced polymers incorporating a hybrid interphase region. Compos. Interfaces.

[38] Papanicolaou G.C., Bouboulas A.S., Anifantis N.K. (2009). Thermal expansivities in fibrous composites incorporating hybrid interphase region. Compos. Struct..

[39] Portan D.V., Papanicolaou G.C. (2018). Properties predictive modeling through the concept of a hybrid interphase existing between phases in contact. AIP Conf. Proc..

[40] Papanicolaou G.C., Baxevanakis C. (1991). Viscoelastic Modelling and Strain-Rate Behaviour of Plasticized Poly (Vinyl Chloride). J. Mater. Sci..

[41] Theocaris P.S., Papanicolaou G.C., Kontou E.A. (1982). The Effect of Filler-Volume Fraction and Strain Rate on Tensile Properties of Iron-Epoxy Particulate Composites. J. Reinf. Plast. Compos..

[42] Papanicolaou G.C., Kontaxis L.C., Manara A.E. (2016). behaviour and modelling of nano and micro TiO2 powder- epoxy resin composite. Cienc. Tecnol. Mater..

